# Unilateral transient high myopization after pediatric strabismus surgery: Observation by anterior segment optical coherence tomography

**DOI:** 10.1016/j.ajoc.2022.101421

**Published:** 2022-02-10

**Authors:** Akihito Yoshimura, Manabu Miyata, Yuki Muraoka, Kentaro Kawai, Akitaka Tsujikawa

**Affiliations:** Department of Ophthalmology and Visual Sciences, Kyoto University Graduate School of Medicine, Kyoto, Japan

**Keywords:** Anterior segment optical coherence tomography, Strabismus surgery, Myopization, Ciliary detachment

## Abstract

**Purpose:**

To describe an unusual case of unilateral transient high myopization after pediatric strabismus surgery.

**Observations:**

A 6-year-old girl with intermittent exotropia had undergone strabismus surgery and experienced a transient decrease in best-corrected visual acuity (BCVA, from 20/20 to 20/33) with high myopization (from +0.25 D to −9.00 D). Slit-lamp microscopy showed anterior chamber inflammation and a narrow angle only in the affected eye. Anterior segment optical coherence tomography (AS-OCT) revealed ciliary body detachment, a narrow angle, a shallow anterior chamber, and an anteroposterior elongation of the crystalline lens in the affected eye. Her symptoms of blurry vision and high myopization improved 8 weeks postoperatively. AS-OCT revealed the resolution of the ciliary body detachment, an open angle, and a deep anterior chamber, with normalization of the anteroposterior length of the crystalline lens. Her BCVA recovered to 20/20 with spherical equivalence of 0 D.

**Conclusions and Importance:**

We reported on an extremely rare case of unilateral transient high myopization following strabismus surgery, which was resolved without additional surgery. This myopization was probably caused due to ciliary body inflammation.

## 1 Introduction

Strabismus surgery has been reported to change refractive errors.^1^, ^2^, ^3^, ^4^ The mean changes in the spherical equivalent refraction and cylinders are small and clinically irrelevant (0.23 ± 0.78 diopters [D] and 0.072 ± 1.22 D, respectively).^3^ Furthermore, the myopic shift resolves 4–6 months after the surgery. To our knowledge, a clinically significant change of refraction after strabismus surgery has not been reported.

Rectus muscle surgery sometimes induces transient ischemia of the anterior segment because it cuts off blood supply to the anterior uvea from the anterior ciliary artery along the rectus muscle.^5^, ^6^, ^7^ In grade 3 anterior segment ischemia, uveitis is observed.^7^ Some case reports showed bilateral moderate myopization with uveitis.^8^^,^^9^ Rectus muscle surgery has a possibility of causing myopization. Thus, rectus muscle surgery has the possibility of causing myopization.

Anterior segment optical coherence tomography (AS-OCT) is a useful modality for the assessment of anterior segment anatomy even in the field of strabismus.^10^ Here, we report a case of unilateral transient high myopia with a decrease in best corrected visual acuity (BCVA) associated with ciliary body detachment and a change in the crystalline lens documented using AS-OCT following strabismus surgery.

## 2 Case report

A 6-year-old girl presented to an outside hospital with intermittent exotropia. She did not have any past medical history aside from febrile seizures at 1 year of age. Her BCVA was 20/20, measured using the Landolt C chart, corrected with a spherical equivalence of +0.25 D. Ocular deviation in primary position was an exodeviation of 35 Δ and 16 Δ at far and near vision, respectively. There was no intraocular inflammation or fundus abnormalities before surgery. The patient underwent lateral rectus recession of 6.0 mm and medial rectus resection of 6.5 mm for the right eye under general anesthesia at the same outside hospital. The surgeon employed 5-0 silk sutures at the upper and lower limbus for traction and used 6–0 vicryl sutures for fixation between the muscle and sclera. No complications, including scleral or corneal perforation, occurred during the surgery. Eye drops of 0.5% levofloxacin and 0.1% betamethasone were administered four times per day after the surgery. One week after surgery, her BCVA decreased to 20/33 with a spherical equivalence of −9.00 D (Table 1). Of note, the patient was diagnosed with influenza 1 month prior to surgery and hemolytic streptococcus 2 weeks following surgery. Both of these illnesses were treated with medication (exact drug could not be determined).

**Table 1:** Changes in the refractive error, lens thickness, and anterior chamber depth in the operated eye.

	Refractive error, diopters (Spherical equivalent)	Lens thickness, mm	Anterior chamber depth, mm
1 month before surgery	+0.25	N/A	N/A
1 week after surgery	−9.00	N/A	N/A
5 weeks after surgery (first visit to our hospital)	−5.50	4.216	1.955
7 weeks after surgery	−5.50	4.006	2.133
8 weeks after surgery	0	3.498	2.950
11 weeks after surgery	−0.50	N/A	N/A

N/A, not available.

Five-week refractive-error, lens thickness, and anterior chamber depth in the fellow eye were 0 diopter, 3.528 mm, and 3.007 mm, respectively.

Since her blurry vision and high myopization did not improve after 4 weeks of follow up, she was referred to our hospital. Eye drops of 0.1% bromfenac sodium hydrate were administered for 3 days between the final visit to that outside hospital and the first visit to our hospital.

**Fig. 1:**
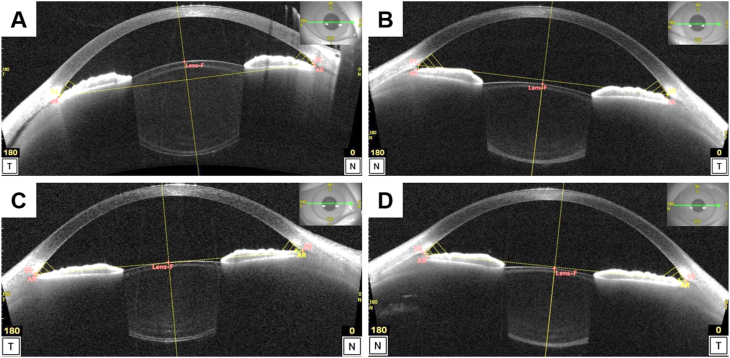
Anterior segment optical coherence tomography images taken 5 and 8 weeks after surgery. Anterior segment optical coherence tomography images acquired 5 weeks (A, B) and 8 weeks (C, D) after surgery. (A, C) The affected eye (right eye) images. (B, D) The fellow eye (left eye) images. (A) The ciliary body detachment, narrow angle, shallow anterior chamber (1.955 mm), and anteroposterior elongation of the crystalline lens (4.216 mm) can be observed along with the fellow eye (B, 3.007 mm and 3.528 mm, respectively). The best-corrected visual acuity (BCVA) was 20/25 with a spherical equivalence of −5.50 D. (C) The disappearance of the ciliary body detachment, an open angle, a deep anterior chamber (2.950 mm), and normalization of anteroposterior length of the crystalline lens (2.950 mm) were observed. The BCVA recovered to 20/20 with a spherical equivalence of 0 D.

**Fig. 2:**
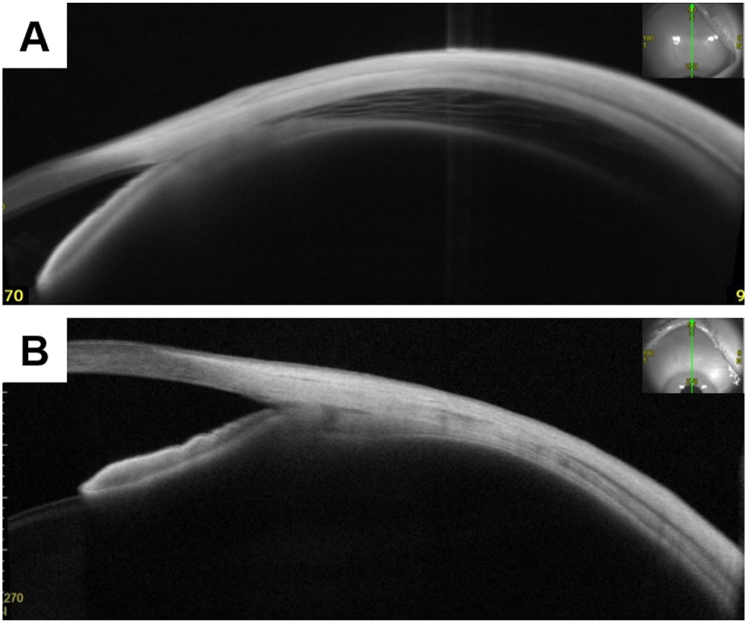
Wide-angle superior anterior segment optical coherence tomography images acquired 5 and 8 weeks after surgery. Ciliary and choroid detachment was observed 5 weeks after surgery (A); however, it disappeared 8 weeks after surgery (B). The findings were observed in the whole circumference.

**Fig. 3:**
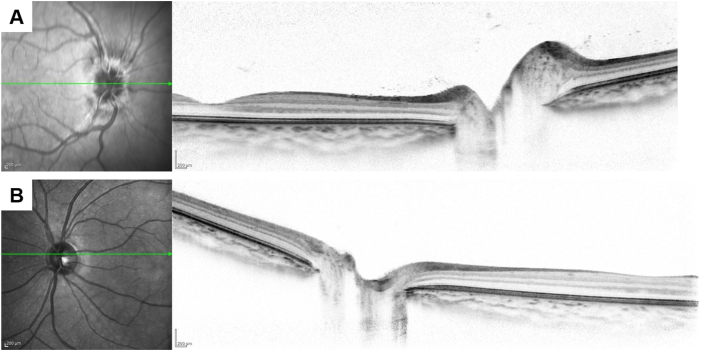
Spectral domain optical coherence tomography fundus images taken 5 weeks after surgery. Optic disc edema and vitreous flare were observed in the affected eye (A) but not in the fellow eye (B).

Upon arrival at our hospital, 5 weeks postoperatively, her BCVA and spherical equivalence were 20/25 and −5.50 D in the affected eye and 20/17 and −0.25 D in the fellow eye. Intraocular pressure measured using a noncontact tonometer was 7 and 18 mmHg in the affected and fellow eyes, respectively. Slit-lamp microscopy revealed inflammation and cells in the anterior chamber and narrow angle only in the affected eye. No corneal perforation was observed. AS-OCT revealed detachment of the ciliary body, a narrow angle, shallow anterior chamber (affected eye vs. fellow eye, 1.955 mm vs. 3.007 mm), and an anteroposterior elongation of the crystalline lens (affected eye vs. fellow eye, 4.216 mm vs. 3.528 mm) (Fig. 1). Anterior dislocation of the crystalline lens was also observed in the affected eye. The axial length of the affected and fellow eye measured using partial coherence interferometry was 22.30 mm and 22.75 mm, respectively. Ciliary and choroid detachment was observed using wide-angle AS-OCT images (Fig. 2). The findings were observed in the whole circumference. Furthermore, corneal or scleral perforation were not detected using AS-OCT to the extent observed. Optic disc edema and anterior chamber and vitreous flare were observed only in the affected eye using slit-lamp microscopy and OCT (Fig. 3). After the first visit to our hospital, eye drops of 0.1% betamethasone were administered 4 times per day.

Her symptoms of blurry vision and high myopization improved 8 weeks postoperatively. AS-OCT revealed resolution of the ciliary body detachment, an open angle, and a deep anterior chamber (2.950 mm), with normalization of the anteroposterior length of the crystalline lens (3.498 mm). Her BCVA recovered to 20/20 with spherical equivalence of 0 D. Slit-lamp microscopy revealed no anterior chamber inflammation or cells. Intraocular pressure was 19 and 15 mmHg in the affected and fellow eyes, respectively. Intraocular pressure of the right eye was slightly increased although it was within the normal limit. Since we considered the administration of 0.1% betamethasone to have caused this increase, and the uveitis resolved, we changed the eye drops to 0.1% fluorometholone three times per day. The patient was orthophoric in primary position. Recurrence of myopization was not detected 11 weeks postoperatively. Optic disc edema was determined to be resolved through observation using fundus OCT. The patient resumed follow up locally at the original outside hospital.

## 3 Discussion

Our patient experienced unilateral transient high myopization with anteroposterior elongation and anterior dislocation of the crystalline lens following strabismus surgery, although previous reports showed clinically irrelevant transient small changes of refractive errors.^1^, ^2^, ^3^, ^4^ The flare of the anterior chamber and vitreous, optic disc edema, ciliary and choroid detachment following surgery, absence of blurry vision prior to the surgery, unilaterality of myopization, and absence of perforation of the cornea and sclera suggest uveitis due to the strabismus surgery. We speculate that uveitis induced the ciliary and choroid detachment, the relaxation of the Zinn's zonule, and anteroposterior elongation and anterior dislocation of the crystalline lens. Consequently, this could have led to the transient high myopia and decreased BCVA. Previous case reports have shown bilateral moderate myopization in adults with uveitis, which was resolved.^8^^,^^9^ In the present study, severe myopization occurred. The cause of the difference in severity might lie in the flexibility of the pediatric crystalline lens. The anteroposterior elongation might be easily induced in a child.

Transient anterior segment ischemia due to recession and resection might induce inflammation. Strabismus surgery has been reported to induce transient ischemia of the iris and episclera, as observed using AS-OCT angiography.^5^^,^^6^ Another previous study reported that anterior segment ischemia after rectus muscle surgery in adults required a full 2–12 weeks of recovery after surgery.^7^ They mentioned that “postoperative uveitis” was observed with grade 3 ischemia. However, this case had low risk of anterior segment ischemia because of the patient's young age; lack of systemic disease including hypertension and atherosclerosis; and a history of only two rectus muscle surgeries. However, we should consider that anterior segment ischemia and subsequent uveitis may occur after recession and resection of the extraocular muscles even in a young patient who does not have any risk factors.

## 4 Conclusion

We reported on an extremely rare case of unilateral transient high myopization following strabismus surgery, which was resolved without additional surgery. This myopization was probably caused due to ciliary body inflammation.

## Institutional review board approval statement and patient consent statement

All study protocols adhered to the tenets of the Declaration of Helsinki. Although ethical guidelines provided by Ministry of Health, Labour and Welfare; and the Ministry of Education, Culture, Sport, Science and Technology in Japan allow a waiver of informed consent for case reports, this study candidate agreed to participate after providing informed consent.

## Funding

This work was supported in part by a grants-in-aid for scientific research (no. 18K09444 and 21K09716) from the 10.13039/501100001691Japan Society for the Promotion of Science, Tokyo, Japan. This organization had no role in the design or conduct of this research.

## Authorship

All authors attest that they meet the current ICMJE criteria for authorship.

## Declaration of competing interest

The authors declare the following financial interests/personal relationships that may be considered as potential competing interests: AY and KK: None. MM: Alcon Japan (F), Novartis Pharma (F), Santen Pharmaceutical (R), and HOYA (R). YM: Bayer Yakuhin (F and R), Novartis Pharma (F and R), Canon (R), Santen Pharmaceutical (R), Senju Pharmaceutical (R), and NIDEK CO, LTD (R). AT: Canon (F and R), Findex (F), Santen Pharmaceutical (F and R), Kowa Pharmaceutical (F), Pfizer (F and R), AMO Japan (F and R), Senju Pharmaceutical (F, R, and C), Wakamoto Pharmaceutical (F and R), Alcon Japan (F, R, and C), Alcon Pharma (F, R, and C), Otsuka Pharmaceutical (F and R), Taiho Pharma (F), Tomey Corporation (F), Bayer Yakuhin (R and C), Novartic Pharma (R), Sanwa Kagaku Kenkyusho (R), Nitten Pharmaceutical (R), AbbVie GK (R), HOYA (C), Janssen Pharmaceutical (C), Kyoto Drug Discovery & Development (C), Daiichi-Sankyo (C), Chugai Pharmaceutical (C), Novartis Pharma AG (C), Eisai (C), Astellas (C), Allegan Japan (C), MSD (C), and Ellex (C).
